# Suicidal Ideation, Lifestyle Factors, and Burnout Syndrome Among Spanish Professionals in Implant Dentistry: A Survey-Based Cross-Sectional Observational Study

**DOI:** 10.3390/jcm14155486

**Published:** 2025-08-04

**Authors:** Ángel-Orión Salgado-Peralvo, Naresh Kewalramani, Eugenio Velasco-Ortega, José López-López, Álvaro Jiménez-Guerra, Loreto Monsalve-Guil, Jesús Moreno-Muñoz, José-Luis Rondón-Romero, Iván Ortiz-García, Enrique Núñez-Márquez

**Affiliations:** 1 Department of Surgery and Medical-Surgical Specialties, Faculty of Dentistry and Medicine, University of Santiago de Compostela, 15782 Santiago de Compostela, Spain; 2 Department of Nursing and Stomatology, Faculty of Dentistry, Rey Juan Carlos University, 28008 Madrid, Spain; k93.naresh@gmail.com; 3 Department of Stomatology, Faculty of Dentistry, University of Seville, 41009 Seville, Spain; evelasco@us.es (E.V.-O.); alopajanosas@hotmail.com (Á.J.-G.); lomonsalve@hotmail.es (L.M.-G.); jolurr001@hotmail.com (J.-L.R.-R.); ivanortizgarcia1000@hotmail.com (I.O.-G.); enrique_aracena@hotmail.com (E.N.-M.); 4 Department of Odontostomatology, Faculty of Dentistry, University of Barcelona, 08907 Barcelona, Spain; 18575jll@gmail.com

**Keywords:** burnout, psychological, burnout, professional, caregiver burden, the Maslach Burnout Inventory, dental implants, surveys and questionnaires, suicide, suicide prevention

## Abstract

**Background**: Burnout syndrome (BS) is an occupational phenomenon resulting from chronic workplace stress that has not been successfully managed. Although there are underlying causes associated with personal attributes, it is generally linked to external factors within the work environment. The aim of the present study was to investigate the impact of lifestyle factors on BS and its dimensions, as well as on suicidal ideation among Spanish professionals dedicated to implant dentistry. **Methods**: A cross-sectional observational study was conducted in accordance with the Strengthening the Reporting of Observational Studies in Epidemiology (STROBE) guidelines. An electronic survey based on the Maslach Burnout Inventory—Human Services Survey (MBI–HSS) was distributed to members of the Spanish Society of Implants. The data were analysed using descriptive statistical methods. **Results**: A total of 305 participants (20.9%) responded to the questionnaire. Notably, 10.8% of the professionals reported experiencing suicidal thoughts, a factor significantly associated with the presence of BS. The lifestyle factors associated with BS included the following: not engaging in aerobic exercise for at least 30 min per day (*p* < 0.05), not having hobbies that facilitate mental disconnection from work (*p* < 0.001), not following a balanced diet (*p* < 0.0001), having an insufficient social life (*p* < 0.0001), and experiencing suicidal ideation (*p* < 0.01). **Conclusions**: The surveyed dentists generally reported having healthy lifestyle habits. Nevertheless, one in ten professionals acknowledged having experienced suicidal ideation at some point, highlighting a concerning association with BS.

## 1. Introduction

Burnout syndrome (BS) is an occupational phenomenon resulting from chronic workplace stress that has not been successfully managed. It is characterized by three dimensions: (1) feelings of energy depletion or emotional exhaustion (EE); (2) increased mental distance from one’s job or feelings of negativism or cynicism related to one’s job—i.e., depersonalization (DE); and (3) reduced professional efficacy and low personal accomplishment (PA) [1]. Although underlying causes may be linked to personal traits, BS is usually associated with external factors specific to the work environment [2].

In this regard, dentistry is widely recognized as a highly stressful profession [3], as several studies have shown that dentists are significantly more likely to experience BS symptoms than professionals in other medical fields [4,5]. This may be partly due to the profound socio-occupational changes that the profession has undergone in the past decade, such as an increase in the number of new graduates and the emergence of new employment conditions, including multiple job holdings. In fact, a recent study conducted in Spain revealed that 64.3% of implant dentistry professionals work in more than one practice [6], a phenomenon also observed in countries like Colombia, where dental specialists work, on average, in three different clinical settings [7]. Additionally, new types of contracts and collaborative models have emerged, including business models based on franchises and insurance companies, along with a growing demand for further specialization [8]. This situation in Spain can largely be attributed to a high level of competitiveness, with a dentist-to-population ratio nearly three times higher than that recommended by the World Health Organization (WHO) (2.94 dentists per 1000 population versus vs. the recommended 1 per 3500 population) [9]. Projections for the coming years do not indicate a more favourable outlook, with recent Eurostat data estimating a national average of 3.62 dental graduates per 100,000 inhabitants (ranking 8th in Europe)—above the European mean of 3.20 [10]. As a result, an increasing number of Spanish dentists are overqualified while facing stagnant or deteriorating working conditions, a combination that may foster frustration and anxiety.

Other factors inherent to the dental profession include the isolated nature of the dentist in their workplace [7,11], pressure to meet professional and occupational goals [7], and long working hours [7,12], as dentists commonly work split shifts and frequent appointment delays often result in limited free time. In addition, there are operational factors, such as the need for great manual precision, a noisy medical environment, difficult work postures, prolonged surgical procedures [13], and extended periods of concentration—which increase the perception of both physical and mental fatigue [11]—limited operating space, and a constant pursuit of technical perfection [13]. Ultimately, dentists are required to work with the precision of a watchmaker in a biological setting involving fluids, muscles, and a patient who is, in turn, another source of stress, as up to a quarter of them present as anxious, critical, uncooperative, or display interrogative and/or obsessive-compulsive behaviours, thereby requiring special management techniques [14], all within a limited time for each visit [7]. Given that dental care is not included in the portfolio of services covered by the Spanish National Health System, patients must bear the associated costs, which are sometimes considerable. Consequently, they demand precise and predictable treatments while not always appreciating the quality of care received [3]. Furthermore, many patients are unable to undergo necessary procedures for financial reasons, which clashes with the perfectionist tendencies of many dentists, leading to demoralization and frustration due to the inability to provide the best possible treatment [11]. This situation is compounded by the ongoing need for continuous professional development due to constant advances in techniques, materials, and technologies [11,15], as well as the existence of negative public perceptions of dentists [11,16]. Yet another factor to consider is the increasing regulatory burden and growing fiscal pressures [3].

This multifactorial context increases the risk of experiencing BS, placing individuals at increased risk of potentially serious personal and professional consequences [2]. Such consequences heighten the likelihood of developing mental health disorders that negatively impact the professional’s quality of life and, secondarily, may lead to family breakdown, alcoholism, or suicide. Additionally, somatization may occur, manifesting as a range of conditions from functional disorders to cardiovascular events, stroke, insomnia, hypertension, and musculoskeletal disorders [17,18].

Therefore, the aim of the present study is to investigate how lifestyle factors influence BS and its dimensions—EE, DE, and PA—as well as their association with suicidal ideation among Spanish professionals dedicated to implant dentistry.

## 2. Materials and Methods

### 2.1. Study Design

A cross-sectional observational study was conducted following the Strengthening the Reporting of Observational Studies in Epidemiology (STROBE) guidelines [19]. The study adhered to all applicable laws and institutional guidelines and was approved by the ethics committee of the CEIm San Carlos Clinical Hospital (Madrid, Spain) (approval no. 24/280-E; 12 April 2024).

### 2.2. Hypothesis

Spanish professionals dedicated to implant dentistry exhibit a rate of suicidal ideation similar to that reported in other studies.

### 2.3. Questionnaire

The survey was distributed via Google Drive to all members of the Spanish Society of Implants (Sociedad Española de Implantes (SEI)) who had not opted out of receiving emails (n = 1460). The survey remained open from May to December 2024, during which 4 reminder emails were sent to maximize response rates. Completion of the survey constituted informed consent for data collection. The final sample included professionals who fully completed the questionnaire. Each participant was allowed to complete the survey only once. No incentives were provided for participation.

A low or limited risk of bias was anticipated due to the similarity between the demographic, academic, and professional characteristics of the participants compared with the rest of the members of the SEI, as well as the broad dissemination of the survey among them.

The questionnaire consisted of three mandatory sections (Table S1), structured so that participants could not proceed to the next question without answering the previous one. The first two sections were analysed in the initial part of the present study [20]. Section 1 examined the characteristics of the surveyed professionals, including demographic, academic, and professional data. Section 2 employed the Maslach Burnout Inventory—Human Services Survey [21] (MBI—HSS), a validated questionnaire that assesses (1) EE, defined as the experience of feeling emotionally drained by work demands; (2) DE, referring to the degree to which individuals acknowledge attitudes of emotional detachment and coldness toward their work or patients; and (3) PA, reflecting feelings of self-efficacy and personal achievement at work. The presence and severity of BS among respondents were determined based on scores assigned to each MBI—HSS dimension. High scores on the first two dimensions (EE ≥ 27; DE ≥ 10) combined with low scores on the third (PA ≤ 33) define the syndrome. Moreover, the severity of BS is considered to decrease or increase when indications of BS are present in one or two dimensions, respectively (Table 1).

Finally, Section 3, which is the focus of the present study, comprised twelve questions exploring respondents’ habits and lifestyle factors that could potentially exacerbate or alleviate BS symptoms, as well as their association with suicidal ideation.

### 2.4. Clinical Relevance

It is essential to understand how various lifestyle-related factors affect the three dimensions of BS (EE, DE and PA), as well as on BS itself and suicidal ideation. This knowledge enables the establishment of targeted recommendations for professionals currently experiencing BS and helps prevent its development in those at risk.

### 2.5. Sample Size Calculation

The target population consisted of all members of the SEI (n = 1460 members). Considering a statistical power of 90%, a confidence level of 95%, and a margin of error of 5%, a minimum sample size of 305 respondents was required to detect significant differences.

### 2.6. Statistical Analysis

The collected data were analysed using IBM^®^ SPSS Statistics v.26 software (IBM^®^ Corp., Armonk, NY, USA). Descriptive statistics were used to report the general results of the study. For analyses involving quantitative variables, a normality test was conducted, revealing that none of the variables followed a normal distribution; therefore, non-parametric tests were applied. The Mann–Whitney U test was used for comparisons involving dichotomous variables, and the Kruskal–Wallis test was used for variables with more than two categories. When the latter was significant, pairwise comparisons using the Mann–Whitney U test were performed to identify the groups responsible for the differences. For associations between qualitative variables, the Chi-square test was conducted. To determine which groups contributed to the differences, Haberman’s standardized residuals were employed, obtaining significance for each cell independently. A *p*-value < 0.05 was considered statistically significant.

## 3. Results

The survey was completed by a total of 305 participants, yielding a response rate of 20.9%, which was deemed adequate. The results obtained from Blocks I and II can be consulted in the first part of the study, in which a BS prevalence rate of 4.3% was reported [20].

### 3.1. Descriptive Data

The majority of respondents engage occasionally or frequently in activities such as thinking, reflecting, or meditating (33.8% and 32.8%, respectively). They occasionally share their stressors with others (39.3%) and perform aerobic exercise for at least 30 consecutive minutes, 3 to 5 times per week (28.9%) or occasionally (28.5%), although a similar proportion rarely or never exercises (24.3%).

More than half of the professionals always (19.7%) or almost always (34.8%) listen to the signals their body provides (symptoms, illnesses, etc). Additionally, 56.7% take vacations lasting 3 to 4 weeks annually, and 52.8% engage in hobbies on a weekly basis that allow them to disconnect from work. In this regard, 65.6% of respondents never or rarely engage in conversations or activities related to dentistry outside of work (25.9%) or do so only occasionally (39.7%). Furthermore, 48.2% frequently sleep 7 to 8 h per day, and they always or almost always strive to follow a balanced diet (21.6% and 51.5%, respectively). Regarding social life, 19% describe it as fulfilling, and 44% as sufficient.

Concerning the consumption of substances such as alcohol and/or drugs, most professionals either do not consume them (46.2%) or do so occasionally (38.7%). An alarming finding is that 9.8% of professionals have experienced suicidal thoughts, of whom 1% reported having such thoughts even before starting their professional careers (Table 2).

### 3.2. Lifestyle Factors and BS

The lifestyle factors that were significantly associated with the presence of BS were as follows: not engaging in aerobic exercise for at least 30 min a day (*p* < 0.05), not having hobbies that allow for disconnection from work (*p* < 0.001), not following a balanced diet (*p* < 0.0001), having an insufficient social life (*p* < 0.0001), and experiencing suicidal thoughts (*p* < 0.01).

### 3.3. Suicidal Thoughts

No association was found between suicidal thoughts and the demographic, academic, or professional variables of the respondents, with the exception of two variables. The first pertains to the number of dental implants placed annually, showing that a significantly higher proportion of professionals who place the lowest number of implants (up to 50 per year) had, at some point, contemplated suicide (20.8%; *p* < 0.05). Secondly, the absence of exclusive clinical practice in oral implantology was significantly associated with having experienced suicidal thoughts (11.5%; *p* < 0.05) (Table 3).

Upon analysing the influence of the BS dimensions, significant differences and a linear upward trend were observed in the mean values of EE and DE when comparing respondents who had never experienced suicidal thoughts (18.97% ± 13.18 = low EE; and 4.70% ± 5.67 = low DE) to those who had (31.17% ± 13.87 = high EE; and 7.73% ± 6.30 = average DE) and to those who had them even before starting their professional careers (35.33% ± 15.14 = high EE; and 14.33% ± 11.59 = high DE) (*p* < 0.0001, and *p* < 0.01; respectively), with statistically significant differences between those who had and had not experienced such thoughts (*p* < 0.001, and *p* < 0.0001; respectively). Furthermore, when examining levels of impact, a significant association was found between high EE and high DE and suicidal thoughts (60%, *p* < 0.0001; and 30%, *p* < 0.05; respectively). No differences were observed in the mean PA values among the three groups, as all were within the low range, nor were any differences observed regarding the level of impact (*p* > 0.05) (Table 4 and Table 5).

**Figure 1:**
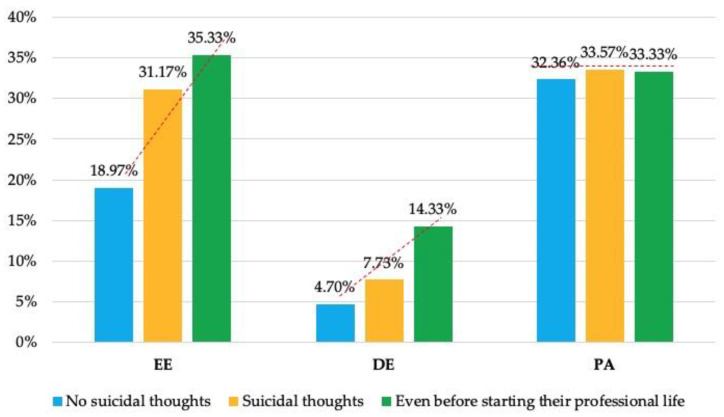
Mean values of BS dimensions (EE, DE, and PA) according to the respondents’ suicidal thoughts.

On the other hand, a significantly higher number of respondents with BS reported having suicidal thoughts (13.3%; *p* < 0.01), as well as having had such thoughts prior to beginning their professional careers (33.3%; *p* < 0.05). It was also observed that the absence of elevated EE or DE values or low PA values was significantly associated with never having experienced suicidal thoughts (*p* < 0.01). Specifically, an ascending linear trend was observed in the values of EE and DE with respect to suicidal ideation status: from never having experienced suicidal thoughts, to having experienced them, and even having experienced them prior to beginning their professional career. No such trend was observed for PA, as values across all three groups remained within the low range (Figure 1).

## 4. Discussion

Lifestyle habits have been associated with mental health and therefore play a key role in the recovery process as well as in the prevention and/or management of BS [22,23], as they lead to physiological and psychological adaptations that facilitate recovery from work and enhance one’s ability to cope with work-related stressors [24,25]. In dentistry, the number of studies exploring the association between BS and different lifestyle and relaxation variables remains limited [26], which underscores the relevance of the present investigation. The development of BS follows a sequence of three progressive stages: initially, a high level of EE, followed by high DE, and eventually, a low sense of PA. For this reason, it is essential for professionals to complete the MBI—HSS [21] on a regular basis, not only to detect BS once it is fully established, but also to diagnose it at earlier stages. This would enable targeted interventions based on the specific dimensions affected, aiming to restore them to optimal levels.

In this regard, factors significantly associated with low EE include (1) taking 3–4 weeks of vacation each year (63.6%; *p* < 0.01), whereas never taking vacations is associated with high EE (50%; *p* < 0.01), and (2) engaging in aerobic physical activity for at least 30 consecutive minutes, 3–5 times per week (71.6%; *p* < 0.001). Several authors have associated physical activity with a reduced risk of BS [25,27], as it serves as a distraction from work-related problems, enhances resilience to stress by improving mood [27], and generally contributes to improved physical health and overall wellbeing [28]. One study revealed that dentists with high levels of stress and BS also reported lower satisfaction with their general health status [3]. Similarly, a systematic review found that being physically active 2–5 times per week for 20–60 min over a period of 18 weeks was associated with a 6.9% to 41.3% reduction in the risk of BS and/or its individual dimensions [24]. Rarely or never engaging in physical activity is associated with high EE (39.2%; *p* < 0.05), as a sedentary lifestyle reduces mood, motivation, and active engagement in rewarding activities [29], thereby increasing the risk of developing BS [30,31]. Lack of time (72.7%) and fatigue (40.8%) were identified as the main barriers to physical activity, with a more pronounced impact on women compared with men (*p* = 0.034 and *p* = 0.003, respectively) [32].

Other factors include (3) engaging in hobbies that allow for weekly disconnection from work (64.6%; *p* < 0.01), such as listening to music [26], whereas not having hobbies or practicing them only occasionally leads to high EE (48.5%; *p* < 0.01), or (4) consistently listening to the messages the body conveys (70.0%; *p* < 0.05). In this regard, one study reported the highest levels of depression and anxiety among professionals suffering from BS (10.4% and 16.2%, respectively), followed by those with high EE and those who rated their health as poor or fair (27.1% and 19.6%, respectively), compared with respondents without BS (5.7%). A similar trend has been observed in the prevalence of tinnitus and oral health problems [33]. Stress has been identified as a risk indicator for periodontal and peri-implant diseases in adults [34]. A study conducted in an Italian hospital among 2216 healthcare professionals revealed that, in 35% of cases, high EE was associated with physical tiredness, and in 50% of cases was associated with physical exhaustion, while more than 30% reported still feeling tired upon waking and facing a new workday [35]. Other signs may include neck and back pain, sleep disturbances, and memory problems [33]. Nevertheless, performing any kind of exercise is associated with fewer physical complaints, such as tension headaches (*p* = 0.026), lower back pain (*p* = 0.015), feeling exhausted, weak or lethargic (*p* < 0.001), and having a fast or irregular heartbeat (*p* = 0.009) [32].

It is also important to (5) regularly sleep 7–8 h per night (62.6%; *p* < 0.05), in line with the recommendations of the U.S. National Sleep Foundation [36], as it is a crucial factor to reduce stress and fatigue after a busy day [26]. During sleep, the body carries out a series of vital processes that contribute to the restoration and strengthening of physical and mental health [37]. Other authors have also reported significantly higher levels of BS among healthcare workers who sleep fewer than 7 h per day (*p* < 0.0001), with these individuals being 12.46 times more likely (95% CI = 8.11–16.80) to experience BS compared with those who sleep more than 7 h, and with women affected more than men (17.18 [95% CI = 10.50–23.87] and 8.33 [95% CI = 2.68–13.99], respectively) [38]. Importantly, not only the quantity but also the quality of sleep is key to preventing BS [39]. Never or almost never achieving this amount of rest is associated with high EE (45.6%; *p* < 0.01).

Other factors associated with lower EE are (6) shorter commuting time to work, (7) satisfaction with salary [7], and (8) having a fulfilling (69.9%; *p* < 0.05) or adequate (65.4%; *p* < 0.01) social life, whereas longing for more social interaction is associated with high EE (48.8%; *p* < 0.00001). In this regard, Slabšinskienė et al. [26] have observed that spending time with family or friends positively influences EE.

Moreover, several factors have been linked to lower levels of DE. Some overlap with those previously mentioned for EE, such as (1) taking 3–4 weeks of annual vacation (78.6%; *p* < 0.0001), whereas never taking time off was associated with high DE (31.3%; *p* < 0.01); (2) enjoying a fulfilling social life (81.0%; *p* < 0.05), while perceiving it as insufficient was associated with high DE (29.6%; *p* < 0.05); and (3) engaging weekly in hobbies that provide mental detachment from work (75.2%; *p* < 0.05), a factor widely recognized as protective against BS [40,41]. Conversely, lacking such hobbies or engaging in them only occasionally was found to be significantly associated with high EE (39.4%; *p* < 0.0001). Additional important factors include (4) dedicating time daily to meditate, reflect, or think (82.6%; *p* < 0.05), as doing so was found to be rarely or never linked to high DE (26.8%; *p* < 0.01), and (5) consistently adhering to a balanced diet (81.8%; *p* < 0.01). A recent systematic review identified fruit and vegetable consumption, along with good overall diet quality, as protective factors against psychological distress [42]—specifically, showing inverse associations with depression and anxiety [43]—as well as against BS [44]. These effects may be mediated by changes in brain-derived neurotrophic factor levels, which are often diminished in individuals with BS symptoms [45] and are influenced by dietary intake [46,47]. Other studies addressed the issue from a reverse perspective, reporting correlations between fast food consumption and BS [31] due to its adverse impact on mood [48]. Consistent with these findings, the present study observed that frequent consumption of “junk food” was found to be associated with high DE (75.0%; *p* < 0.001). Nevertheless, some authors have found that greater fast-food consumption and lower fruit and vegetable intake are associated with lower EE but higher DE [49]. Slabšinskienė et al. [26] have also reported that any form of (6) passive rest, except regular watching TV, had a favourable impact on DE levels.

Finally, lifestyle factors associated with high PA are (1) frequently sleeping 7–8 h per night (51.7%; *p* < 0.05), as never or almost never doing so leads to low PA (31.6%; *p* < 0.05); (2) meditating frequently (56%; *p* < 0.01) or occasionally (35.9%; *p* < 0.05); and (3) consuming alcohol or drugs at least once a week, which is associated with low PA (68.4%; *p* < 0.05). A recent study has reported that one in five dental professionals (17.4%) engage in a potentially hazardous or risky consumption of alcohol [16]. In the UK, 22.1% of dentists drink alcohol four or more times per week and 5.6% daily [3], prompting the question of “which comes first, the chicken or the egg,” or in other words, whether it is cause or consequence, as BS often coexists with negative emotions that lead to negative lifestyle choices [50,51]. Several studies have examined the link between BS and alcohol and tobacco consumption with contradictory results [33,34,35,36]. A survey conducted in Lithuania has shown that professionals who regularly consume illegal substances, tobacco, alcohol, or medications to reduce stress or improve sleep presented 1.14 to 1.40 times higher levels of EE compared with those who do not consume these substances, with significant differences that also negatively affect DE. Furthermore, multivariate analyses demonstrated a relationship between tobacco consumption and significantly higher DE (95% CI = 0.22–4.62; *p* = 0.031) and lower PA (95% CI = 0.29–4.27; *p* = 0.025) [7], while other studies have reported a negative impact on all dimensions of BS, thus considering tobacco consumption as a predictor of feelings of stress and exhaustion related to work problems [26], potentially serving as an escape route. Another investigation showed that tobacco consumption is associated with odds ratios (OR) of 2.92 (*p* = 0.0365) for high EE versus low EE and 2.50 (*p* = 0.0343) for low PA versus high PA, whereas alcohol consumption showed an OR of 4.67 (*p* < 0.0001) for high DE versus low DE [35]. Smoking helps smokers cope with negative affective states as nicotine exerts a greater effect in stressful situations, which explains its addictive potential in individuals with high levels of anxiety, stress, or depression [52].

On the other hand, alcohol may be used as a relaxant and a means of escape [53,54], though excessive consumption is dangerous due to its association with deaths caused by external factors [55] and with 30.5% of suicides in Finland [11]. In the present study, professionals who consume toxic substances once a week had significantly higher DE levels (9.53 ± 8.23) than those who consumed them occasionally (4.98 ± 5.81; *p* < 0.01) or not at all (4.43 ± 5.22; *p* < 0.01), as well as lower PA levels (24.37 ± 11.50) compared with occasional users (33.92 ± 14.44; *p* < 0.01) or non-users (33.00 ± 14.65; *p* < 0.01). Another factor associated with low PA is (4) watching TV [26], while PA is increased by (5) occasionally continuing to talk or engage in dental-related activities outside of work (37.2%; *p* < 0.05) or never/almost never (57.0%; *p* < 0.05) and (6) by performing some type of non-dental work activity compared with not doing so (38.05 ± 6.05 vs. 35.35 ± 5.05, respectively; *p* = 0.015) [56]. Indeed, engaging in relaxing hobbies, such as regularly attending cultural events, spending time in nature, and reading non-medical literature, has positive effects on all three dimensions of BS [26].

Stress is defined as an adaptive physiological and psychological response to challenges that exceed an individual’s capacity to manage them effectively [57]. In other words, it should be an acute response rather than a chronic condition. Occupational stress in dentistry has been associated with an increased risk of developing mental health problems that worsen over time, such as depression, anxiety, and BS [58]. A recent study has shown that more than one-third of dental professionals present elevated anxiety levels (34%) [59]. Dentists scored lower than the general population regarding life satisfaction (5.7 vs. 7.7, respectively), life worthwhileness (6.3 vs. 7.9), and happiness (5.7 vs. 7.5), while scoring higher on anxiety (5.0 vs. 2.9). These parameters were significantly worse among dentists with BS compared with those without (satisfaction with life = 5.41 vs. 7.72, respectively; *p* < 0.001; worthwhile = 5.86 vs. 8.15; *p* < 0.001; happiness = 5.38 vs. 7.76; *p* < 0.001; and anxiety = 5.39 vs. 2.73; *p* < 0.001) [3].

In the first part of the present study [20], a BS prevalence rate of 4.3% was reported. If left untreated, BS can lead to medical problems such as cardiovascular or musculoskeletal diseases [60], as well as mental health issues including depression [61]. A study conducted in Pakistan observed that 64.1% of dental professionals suffered from depression (mild = 28.9%; moderate = 18.2%; moderately severe = 11.3%; severe = 5.7%) [59], whereas in the United States (U.S.), this prevalence was 25.3% (mild = 20.2%; moderate = 4.4%; severe = 0.7%), with no association found with demographic factors, but a significant relationship with the dimensions of BS—40.8% of professionals presenting high values of EE and DE had moderate-to-severe depression (*p* < 0.05) [61]. Alarmingly, only a very small percentage of dentists with depression (15%) were receiving treatment [62]. U.S. surgeons expressed reluctance to seek care for mental health problems “due to concerns that it might affect their medical license” [63]; other reasons may include normalization of the problem, lack of awareness of its extent, stigmatization, emphasis on maintaining a professional culture, or inadequate support [57]. Other authors observed a significantly higher prevalence of depression among dentists compared with other dental professionals (oral health therapists, dental therapists, dental hygienists, and dental prosthetics) (*p* < 0.001), for females (*p* < 0.001), for those with fewer years of experience (*p* = 0.002), and for those working in private dental clinics (*p* = 0.003) [59]. These data should not be underestimated, as severe depression may culminate in suicide [16,59,64] (OR = 1.62 [95% CI = 1.00–2.61]) [16].

In this regard, the present study described a suicidal ideation rate of 10.8% (n = 33), of whom 1% had such thoughts even before starting their professional career. A relationship with anxiety [16,59], limited work experience [59], high job stress, psychological distress, and BS [3] has also been observed. Furthermore, a previous suicide attempt increases the likelihood of current suicidal ideation compared with those who have never attempted suicide (38.7% [95% CI = 25.7–53.5%] vs. 17.3% [95% CI = 12.7–23.2%], respectively) [16]. According to the literature, there is an average of 8–20 failed attempts for each successful suicide [11]. At the end of the 1970s and the beginning of the 1980s in the UK, dentistry was the profession with the sixth highest suicide rate (35.6% per 100,000 inhabitants). Fortunately, this trend has decreased, declining to 14.6%, representing a reduction of –58.9% between 2001 and 2005 (*p* < 0.05) [65]. In the same country, a 2019 study involving 2053 dentists found that 17.6% reported suicidal ideation (57.7% within the last 12 months), while 9.9% preferred not to answer, suggesting that these figures may be underestimated [3]. A review conducted in 2010 found that the suicide rate among working-age dentists worldwide was 5.43 times higher than that of the general population [11]. A Danish study spanning 26 years (1981 to 2006) observed that healthcare professions (nurses, physicians, pharmacists, and dentists) had a higher risk of suicide than teachers, veterinary surgeons, and the general population, with the highest relative risk (RR) in dentists (RR = 2.61; 95% CI = 1.91–3.56). The suicide rate in dentists was 7.19%, showing a male predominance (i.e., four times higher risk than females). A hopeful finding was a significant annual decrease in risk of 7% (*p* = 0.004). Among dentists who had previously sought psychiatric help, adjusted rate ratios significantly decreased compared with those who had not sought help (1.76 [95% CI = 0.94–3.26] vs. 3.68 [2.53–5.34], respectively) [66]. Another study conducted in the same country from 1980 to 2021 reported a suicide rate of 18% (95% CI = 11.1–24.9) in male dentists and 6.7% (95% CI = 1.3–12.0) in female dentists, ranking dentistry as the fifth highest healthcare profession (among physicians, veterinarians, psychologists, pharmacists, and nurses) for suicide rate in men and sixth in women. Nevertheless, an overall downward trend was observed during this period [67].

In Austria, a study conducted between 1986 and 2020 investigated the suicide mortality rates (SMR) among working-age dentists, physicians, veterinarians, pharmacists, notaries, tax advisors/public accountants, and lawyers. Dentists ranked fourth in SMR within the study, behind (in ascending order) veterinarians, pharmacists, and physicians, with only the former showing a significant increase above the SMR of the general population. Specifically, the SMR for dentists was 0.85 (95% CI = 0.56–1.24), representing 24 suicide deaths out of a total of 224, with a potentially higher risk in females (1.74 [95% CI = 0.81–3.30]) than males (0.68 [0.40–1.07]), without significant differences when compared with the general population. Age-adjusted suicide rates were 16.6% in males and 17.1% in females (ratio = 0.97), whereas in the general population they were 25.1% and 7.5%, respectively (ratio = 3.35). The most frequently used methods among dentists were firearms and hanging, while poisoning was more common among other healthcare professionals [68].

Petersen and Burnett [69] observed that the suicide rates of white male dentists were higher than those of the general U.S. population between 1984 and 1992, in the age ranges of 55 to 59 years (33.4 [95% CI = 14.4–65.8] vs. 32.0, respectively) and 60 to 64 years (47.5 [95% CI = 21.7–90.1] vs. 42.6, respectively), with an SMR of 0.68 (95% CI = 0.52–0.89). A more recent study (2024) surveyed 597 dentists practicing in the U.S., reporting a rate of suicidal ideation of 6.2% [70].

Recently, an Australian study involving 421 dental professionals (oral health therapists, dental therapists, dental hygienists, and dental prosthetists) and 1052 dentists reported that 17.6% of the former group and 29.8% of the latter experienced suicidal ideation within the past year. Furthermore, prior to that period, suicidal thoughts had been reported by 31.4% of dental professionals and 54.7% of dentists. Alarmingly, 5.6% of dental professionals and 7.5% of dentists had attempted suicide [16]. These figures exceed those observed among physicians in the same country, among whom 10.4% experienced suicidal ideation in the last 12 months, 24.8% had prior suicidal thoughts, and 2.3% had attempted suicide [71]. Lastly, the most recent study conducted in Pakistan surveyed 636 dental professionals (dentists, dental hygienists, dental assistants, and dental prosthetists) and found a suicidal ideation rate of 11.9% [59].

Only 39% of dental professionals who had experienced suicidal ideation sought professional help beforehand [72]. The impact of such ideation on professionals depends, among other factors, on their personality traits and their “locus of control,” that is, the way in which they cope with stress. Several studies have identified specific traits among dental professionals [16,59], showing a high proportion of moderate (11.9%) or highly perfectionist individuals (87.3%), and low levels of resilience (36%). High resilience has been associated with a decreased OR for suicidal ideation (OR = 0.92; 95% CI = 0.89–0.96) [16]. It has been suggested that, on one hand, the chosen profession may shape one’s personality, but on the other, a profession may also attract certain personality types [11]. In this regard, dentistry is thought to attract students with compulsive personality traits, who often hold unrealistic expectations and highly demanding behavioural patterns, along with a strong need for social recognition and high social status [73,74].

In the present study, other variables—such as the marital status of the respondents—were not analysed, as the questionnaire designed was already extensive. Other authors have reported that single dental professionals exhibited significantly higher rates of suicidal ideation in the 12 months prior to the study (27.3%) or at any time before that period (39%), with 9.3% having attempted suicide [16]. This feeling of “being alone” is not only a risk factor for single individuals, but also for those who are divorced, widowed, or have never married, and may be exacerbated by age [75]. It is important to note that such thoughts are strongly associated with high levels of depression and psychological distress, but not with BS, as pointed out by Hopcraft et al. [16] (2023).

### 4.1. General Recommendations

It is recommended that both university programs and professional dental associations teach stress management strategies, encouraging professionals to seek professional help when their own coping capacities are exceeded. Additionally, it is advised to dedicate time to oneself and to develop a healthy lifestyle that promotes both physical and mental health—that is, a “mens sana in corpore sano”. Finally, it is essential to implement organizational and structural measures to improve the work environment in order to achieve more effective and long-lasting changes [3].

### 4.2. Recommendation for Further Research

It is important to understand how practicing dentistry affects mental health. One way to measure this could be by administering mental health and personality questionnaires to first-year dental students to establish their baseline status and thereby identify any pre-existing psychiatric conditions. These results could then be compared with those from practicing professionals. This approach would help to determine whether dentistry attracts certain personality types and/or whether mental health deteriorates as a consequence of professional practice.

### 4.3. Limitations

This study had several limitations. Due to the nature of the survey, the authenticity of the data supplied by respondents cannot be ascertained, as certain personal enquiries may have been answered subjectively, even if the survey was undertaken anonymously. The data obtained may be underestimated, as there may be a significant number of professionals with BS who are less likely to participate in surveys [76], as well as those with suicidal thoughts due to social stigma associated with mental health issues. Furthermore, the lack of validated scales to assess suicidal ideation complicated comparisons with previously published studies. Although the response rate obtained (20.9%) may be considered low, it is comparable to that obtained in similar surveys included in the first part of the present study [20] (range 9.3% [77] to 71.7% [26]). This lower participation can be attributed to the usual care burden of these professionals and the sensitive nature of some questions in the questionnaire. Nevertheless, the obtained sample adequately reflects the distribution by age, sex, and specialty within the target population, suggesting a reasonable representativeness. Even so, a possible non-response bias cannot be ruled out, and therefore, the results should be interpreted with caution. Future studies with higher participation rates will allow for the confirmation of these findings. Finally, given the cross-sectional observational nature of the study, it is not possible to infer the underlying causes of suicidal ideation reported by one-tenth of the respondents. Despite these limitations, this research represents a large-scale study using validated measures that offers comprehensive insight into lifestyle factors and their influence on BS, as well as suicidal ideation.

## 5. Conclusions

Within the limitations of this study, it can be concluded that, overall, Spanish dentists dedicated to oral implantology who have participated in the survey exhibit generally healthy lifestyles, which is reflected in a low prevalence of BS (4.3%). However, one in ten dentists has experienced suicidal ideation at some point. These suicidal thoughts are associated with the presence of BS, highlighting the importance of regularly completing the MBI—HSS. This allows for the identification of altered dimensions (high EE or DE, or low PA), enabling professionals to make lifestyle changes to improve these conditions and to seek professional support when suicidal thoughts are present, as they are a strong predictor of suicide attempts.

## Figures and Tables

**Table 1:** Reference values of the dimensions evaluated in the MBI—HSS.

Dimension	Low	Average	High	Signs of BS ^4^
EE ^1^	0–18	19–26	27–54	≥27
DE ^2^	0–5	6–9	10–30	≥10
PA ^3^	0–33	34–39	40–48	≤33

^1^ Emotional exhaustion; ^2^ depersonalization; ^3^ personal accomplishment; ^4^ burnout syndrome.

**Table 2:** Lifestyle patterns and health-related behaviours of the participants.

Variables	Frequency	N ^1^	% ^2^
Do you dedicate time to think, reflect, or meditate?	Daily	46	15.1
	Frequently	100	32.8
	Occasionally	103	33.8
	Rarely or never	56	18.4
Do you benefit from sufficient and restful holidays?	Every year	173	56.7
	Some years	54	17.7
	Occasionally	46	15.1
	Rarely or never	32	10.5
Do you engage in aerobic exercise for at least 30 consecutive min ^3^?	3–5 times per week	88	28.9
	Frequently	56	18.4
	Occasionally	87	28.5
	Rarely or never	74	24.3
Do you have hobbies that help you disconnect from work?	Weekly	161	52.8
	Monthly	24	7.9
	Occasionally	87	28.5
	Rarely or never	33	10.8
Do you pay attention to your body’s signals (symptoms, illnesses, etc.)?	Always	60	19.7
	Almost always	106	34.8
	Occasionally	93	30.5
	Rarely or never	46	15.1
Do you share your stressors (problems, needs) with others?	Regularly	71	23.3
	Frequently	74	24.3
	Occasionally	120	39.3
	Rarely or never	40	13.1
Do you sleep well (at least 7–8 h ^4^ per night)?	Frequently	147	48.2
	Occasionally	101	33.1
	Rarely or never	57	18.7
Do you try to follow a balanced diet?	Always	66	21.6
	Almost always	157	51.5
	Not often	78	25.6
	I eat a lot of “junk food”	4	1.3
How would you describe your social life?	Fulfilling	58	19.0
	Adequate	136	44.6
	I wish I had more	84	27.5
	Insufficient	27	8.9
Regarding consumption of toxic substances (alcohol and/or drugs):	I do not consume toxic substances	141	46.2
	I consume them occasionally each week as a means of escaping reality	5	1.6
	I consume them occasionally each month as a means of escaping reality	15	4.9
	I consume them occasionally each year as a means of escaping reality	19	6.2
	I consume them several times per week	118	38.7
	I consume them more frequently than I would like	7	2.3
Since beginning your professional career, have you ever thought that it would be better not to be alive?	Yes	272	89.2
	No	30	9.8
	Yes, even before starting my professional career	3	1.0
Outside the workplace, do you usually talk about or engage in activities related to dentistry (i.e., do you find it difficult to disconnect)?	Always	18	5.9
	Frequently	87	28.5
	Occasionally	121	39.7
	Rarely or never	79	25.9

^1^ Participants; ^2^ percentage; ^3^ minute(s); ^4^ hour(s).

**Table 3:** Prevalence of suicidal ideation according to respondents’ demographic characteristics.

Variable	Specifications	Have You Ever Thought That It Would Be Better Not to Be Alive?						*p*-Value
		No		Yes		Yes, Even Before Starting My Professional Career		
		N ^1^	% ^2^ *	N	% *	N	% *	
Gender	Female	80	83.3	14	14.6	2	2.1	quasi
	Male	192	91.9	16	7.7	1	0.5	
Age (years)	≤30	26	92.9	2	7.1	0	0.0	—
	31–40	79	90.8	7	8.0	1	1.1	
	41–50	67	87.0	10	13.0	0	0.0	
	51–60	32	82.1	5	12.8	2	5.1	
	>60	68	91.9	6	8.1	0	0.0	
Basic university education level	Bachelor’s degree in dentistry (Bologna compliant)	67	88.2	8	10.5	1	1.3	—
	Licentiate in dentistry (pre-Bologna degree)	149	87.1	20	11.7	2	1.2	
	Stomatologist	50	96.2	2	3.8	0	0.0	
	Maxillofacial surgeon	6	100	0	0.0	0	0.0	
Highest implant postgraduate training attained	Master’s degree students	13	81.3	3	18.8	0	0.0	—
	Postgraduate certificates	27	87.1	4	12.9	0	0.0	
	Master’s degree	158	90.8	13	7.5	3	1.7	
	Non-accredited training courses	74	88.1	10	11.9	0	0.0	
Experience placing dental implants (in years)	<5	52	91.2	4	7.0	1	1.8	—
	5–15	72	84.7	12	14.1	1	1.2	
	15–20	47	90.4	4	7.7	1	1.9	
	>20	101	91.0	10	9.0	0	0.0	
Approximate average number of implants placed per year	≤50	41	77.4 ^2^	11	20.8 ^2^	1	1.9	<0.05
	51–100	61	93.8	4	6.2	0	0.0	
	101–200	70	87.5	8	10.0	2	2.5	
	>200	100	93.5	7	6.5	0	0.0	
Exclusive clinical practice in dental implant treatments	Yes	43	100 ^1^	0	0.0 ^1^	0	0.0	<0.05
	No	229	87.4 ^1^	30	11.5 ^1^	3	1.1	
Work environment	Rural	30	85.7	5	14.3	0	0.0	—
	Urban	242	89.6	25	9.3	3	1.1	
How do you perform your activity?	Work for others	82	92.1	7	7.9	0	0.0	—
	Clinic owner	119	88.8	13	9.7	2	1.5	
	Both options	71	86.6	10	12.2	1	1.2	
Multiple workplaces	Yes	173	88.3	21	10.7	2	1.0	—
	No	99	90.8	9	8.3	1	0.9	
Approximate number of h ^3^ worked per week	<16	3	75.0	1	25.0	0	0.0	—
	16–24	19	86.4	3	13.6	0	0.0	
	25–32	53	98.1	1	1.9	0	0.0	
	33–40	101	90.2	10	8.9	1	0.9	
	>40	96	85.0	15	13.3	2	1.8	

^1^ Participants; ^2^ percentage; ^3^ hour(s). Statistical significance: *1 = *p* < 0.05; *2 = *p* < 0.01; *3 = *p* < 0.001; *4 = *p* < 0.0001; *5 = *p* < 0.00001.

**Table 4:** BS levels and number of BS dimensions (EE, DE, and PA) according to respondents’ suicidal thoughts.

Variable	Specifications	No		Yes		Yes, Even Before Starting Professional Career		*p*-Value
		N ^1^	% ^2^	N	%	N	%	
EE ^3^	Low	163	59.9 ^2^	9	30.0 ^2^	1	33.3	<0.001
	Medium	42	15.4	3	10.0	0	0.0	
	High	67	24.6 ^4^	18	60.0 ^4^	2	66.7	
DE ^4^	Low	194	71.3 ^2^	15	50.0 ^1^	1	33.3	<0.01
	Medium	44	16.2	6	20.0	0	0.0	
	High	34	12.5 ^2^	9	30.0 ^1^	2	66.7 ^1^	
PA ^5^	Low	115	42.3	12	40.0	1	33.3	—
	Medium	31	11.4	6	20.0	1	33.3	
	High	126	46.3	12	40.0	1	33.3	
BS ^6^	Positive	8	2.9 ^2^	4	13.3 ^2^	1	33.3 ^1^	<0.01
	Negative	264	97.1 ^2^	26	86.7 ^2^	2	66.7 ^1^	
Number of dimensions	None	102	37.5 ^2^	3	10.0 ^2^	1	33.3	<0.001
	One dimension	132	48.5	19	63.3	0	0.0	
	Two dimensions	30	11.0	4	13.3	1	33.3	
	Three dimensions (positive BS)	8	2.9 ^3^	4	13.3 ^2^	1	33.3 ^1^	

^1^ Participants; ^2^ percentage; ^3^ emotional exhaustion; ^4^ depersonalization; ^5^ personal accomplishment; ^6^ burnout syndrome. Statistical significance: *1 = *p* < 0.05; *2 = *p* < 0.01; *3 = *p* < 0.001; *4 = *p* < 0.0001; *5 = *p* < 0.00001.

**Table 5:** Mean values and standard deviations of BS dimensions (EE, DE, and PA) according to respondents’ suicidal thoughts.

Variable	No		Yes		Yes, Even Before Starting Professional Career		*p*-Value
	Mean	SD ^1^	Mean	SD	Mean	SD	
EE ^2^	18.97	13.18	31.17	13.87	35.33	15.14	<0.0001
	*		*				<0.001
DE ^3^	4.70	5.67	7.73	6.30	14.33	11.59	<0.01
	*		*				<0.0001
PA ^4^	32.36	14.62	33.57	13.38	33.33	16.17	-

^1^ Standard deviation; ^2^ emotional exhaustion; ^3^ depersonalization; ^4^ personal accomplishment. Statistical significance: *1 = *p* < 0.05; *2 = *p* < 0.01; *3 = *p* < 0.001; *4 = *p* < 0.0001; *5 = *p* < 0.00001.

## Data Availability

The data that support the findings of this study are available on from the corresponding author upon reasonable request.

## Supplementary Materials

The following supporting information can be downloaded at https://www.mdpi.com/article/10.3390/jcm14155486/s1, Table S1: Questionnaire used in the present study.

## Abbreviations

The following abbreviations are used in this manuscript:

BS	Burnout syndrome
EE	Emotional exhaustion
DE	Depersonalization
PA	Personal accomplishment
WHO	World Health Organization
STROBE	Strengthening the Reporting of Observational Studies in Epidemiology
SEI	Sociedad Española de Implantes (Spanish Society of Implants)
MBI—HSS	Maslach Burnout Inventory—Human Services Survey
TV	Television
h	Hour(s)
min	Minute(s)
SD	Standard deviation
OR	Odds ratio
UK	United Kingdom
U.S.	United States
r	Spearman test
vs.	Versus
CI	Confidence interval
RR	Relative risk
SMR	Suicide mortality rate
