# Supplementary material for: Suicidal Ideation, Lifestyle Factors, and Burnout Syndrome Among Spanish Professionals in Implant Dentistry: A Survey-Based Cross-Sectional Observational Study
Source: J Clin Med. 2025 Aug 4;14(15):5486. doi: 10.3390/jcm14155486 (PMC12347348; doi:10.3390/jcm14155486)
Supplement: Supplementary file 1 [file jcm-14-05486-s001.zip › jcm-3797882-supplementary.pdf]

Table S1. Questionnaire employed in the present study.

BLOCK I<br>General Questions

1) Gender:
   a) Female.
   b) Male.

2) Age (years):
   a) ≤ 30
   b) 31 – 40
   c) 41 – 50
   d) 51 – 60
   e) > 60

3) Basic university education level.
   a) Bachelor's Degree in Dentistry (Bologna-compliant)
   b) Licentiate in Dentistry (pre-Bologna degree)
   c) Stomatologist
   d) Maxillofacial Surgeon

4) Highest implant postgraduate training attained.
   a) Currently enrolled in a university master's program related to Oral Implantology (oral surgery, oral implantology, or combinations)
   b) University postgraduate degrees related to Oral Implantology
   c) Completed a university master's degree related to Oral Implantology
   d) Non-accredited training courses (clinical stays, commercial company courses, etc.)

5) Experience placing dental implants (years).
   a) ≤ 5
   b) 5 – 15
   c) 15 – 20
   d) > 20

6) Approximate average number of implants placed per year.
   a) ≤ 50
   b) 51 – 100
   c) 101 – 200
   d) > 200

7) Is your clinical practice exclusively dedicated to implant placement?
   a) Yes
   b) No

8) Work environment
   a) Rural
   b) Urban

9) How do you perform your activity?
   a) Work for others
   b) Owner of my own dental clinic
   c) Both options

10) Do you work at multiple workplaces?

a) Yes
b) No

11) Approximate number of hours worked per week:

a) < 16 h
b) 16 a 24 h
c) 25 – 32 h
d) 33 – 40 h
e) > 40 h

BLOCK II
Burnout Questionnaire

EE – Emotional Exhaustion Assessment

Statements	Degree of agreement (Assigned value)
1) I feel emotionally drained from my work.	Never (0)<br>Occasionally per year (1)<br>Once a month or less (2)<br>A few times a month (3)<br>Once a week (4)<br>A few times a week (5)<br>Every day (6)
2) I feel tired at the end of the workday.	
3) When I wake up in the morning and face another workday, I feel fatigued.	
4) I feel that working all day with patients requires a great effort and tires me.	
5) I feel that my work is wearing me down. I feel burned out by my work.	
6) I feel frustrated at work.	
7) I think I work too much.	
8) Working directly with patients causes me stress.	
9) I feel exhausted at work, reaching the limits of my capabilities.	

DE – Depersonalization Assessment

Statements	Degree of agreement (Assigned value)
1) I feel like I treat some patients as impersonal objects.	Never (0)<br>Occasionally per year (1)<br>Once a month or less (2)<br>A few times a month (3)<br>Once a week (4)<br>A few times a week (5)<br>Every day (6)
2) I have become more insensitive to people since practicing as a dentist.	
3) I think this work is emotionally hardening me.	
4) I don't really care about what happens to some of my patients.	
5) I think patients blame me for some of their problems.	

PA – Personal Accomplishment Assessment

Statements	Degree of agreement (Assigned value)
1) I find it easy to understand how my patients feel.	Never (0)<br>Occasionally per year (1)<br>Once a month or less (2)<br>A few times a month (3)<br>Once a week (4)<br>A few times a week (5)<br>Every day (6)
2) I believe I effectively address my patients' problems.	
3) I think my work positively influences my patients' lives.	
4) I feel energetic at work.	
5) I feel that I can easily create a pleasant atmosphere for my patients.	
6) I feel motivated after working with my patients.	
7) I think I achieve many valuable things in this work.	
8) At work, I deal with emotional problems very calmly.	

BLOCK III
Habits and Lifestyle

1) Do you dedicate time to think, reflect, or meditate?
   a) Daily.
   b) Frequently.
   c) Occasionally.
   d) Rarely or never.

2) Do you benefit from sufficient and restful holidays?
   a) Every year.
   b) Some years.
   c) Occasionally.
   d) Rarely or never.

3) Do you engage in aerobic exercise for at least 30 consecutive minutes?
   a) 3 – 5 times per week.
   b) Frequently.
   c) Occasionally
   d) Rarely or never.

4) Do you have hobbies that help you disconnect from work?
   a) Weekly.
   b) Monthly.
   c) Occasionally.
   d) Rarely or never.

5) Do you pay attention to your body's signals (symptoms, illnesses, etc.)?
   a) Always.
   b) Almost always.
   c) Occasionally
   d) Rarely or never.

6) Do you share your stressors (problems, needs) with others?
   a) Regularly.
   b) Frequently.

c) Occasionally

d) Rarely or never.

7) Do you sleep well (at least 7–8 hours per night)?

a) Frequently.

b) Occasionally.

c) Rarely or never.

8) Do you try to follow a balanced diet?

a) Always.

b) Almost always.

c) Not often.

d) I eat a lot of "junk food"

9) How would you describe your social life?

a) Fulfilling

b) Adequate.

c) I wish I had more.

d) Insufficient.

10) Regarding consumption of toxic substances (alcohol and/or drugs):

a) I do not consume toxic substances

b) I consume them occasionally each week as a means of escaping reality.

c) I consume them occasionally each month as a means of escaping reality.

d) I consume them occasionally each year as a means of escaping reality.

e) I consume them several times per week.

f) I consume them more frequently than I would like.

11) Since beginning your professional career, have you ever thought that it would be better not to be alive?

a) Yes.

b) No.

c) Yes, even before starting my professional career.

12) Outside the workplace, do you usually talk about or engage in activities related to dentistry (i.e., do you find it difficult to disconnect)?

a) Always.

b) Frequently.

c) Occasionally.

d) Rarely or never.
